# HIV-1 co-receptor tropism and liver fibrosis in HIV-infected patients

**DOI:** 10.1371/journal.pone.0190302

**Published:** 2018-01-11

**Authors:** Annalisa Saracino, Alessandro Cozzi-Lepri, Milensu Shanyinde, Francesca Ceccherini Silberstein, Silvia Nozza, Antonio Di Biagio, Giovanni Cassola, Giuseppe Bruno, Maria Capobianchi, Massimo Puoti, Laura Monno, Antonella d’Arminio Monforte, for the ICONA Foundation Study

**Affiliations:** 1 Clinic of Infectious Diseases, University of Bari, Bari, Italy; 2 Department of Infection and Population Health, University College London Medical School, Royal Free Campus, London, United Kingdom; 3 Department of Experimental Medicine, University of Tor Vergata, Rome, Italy; 4 Infectious Diseases Department, San Raffaele Scientific Institute, Milan, Italy; 5 Infectious Diseases Department, IRCCS San Martino Hospital, Genoa, Italy; 6 Division of Infectious Diseases, Galliera Hospital, Genoa, Italy; 7 Laboratory of Virology, National Institute for Infectious Diseases "L. Spallanzani", Rome, Italy; 8 Department of Infectious Diseases, AO Niguarda Ca' Granda, Milan, Italy; 9 Clinic of Infectious and Tropical Diseases, Department of Health Sciences, San Paolo H, University of Milan, Milan, Italy; Western Sydney University, AUSTRALIA

## Abstract

**Background:**

*In vitro*, gp120 of both X4 and R5 HIV-1 strains activates human hepatic stellate cells, but if it can promote liver fibrosis *in vivo* is unknown. We aimed to evaluate if patients carrying X4 or R5 strains have a different liver fibrosis (LF) progression over time.

**Methods:**

A total of 1,137 HIV-infected patients in ICONA cohort (21% females, 7% HCV co-infected) with an available determination of HIV-1 co-receptor tropism (CRT), a Fibrosis-4 Index for Liver Fibrosis (FIB-4) <3.25 and at least one-year follow-up were included. CRT was assessed by gp120 sequencing on plasma RNA and geno2pheno algorithm (10% false positive rate) or by Trofile. LF was assessed by means of FIB-4. LF progression was defined as an absolute score increase or a transition to higher fibrosis stratum and/or occurrence of liver-related clinical events.

**Results:**

A total of 249 (22%) patients carried X4 strains, which were associated with older age, lower CD4 count, lower nadir CD4, and intravenous drug use. Overall, X4 and R5 patients had similar baseline FIB-4 scores and similar mean FIB-4 slope after a median follow-up of 35 months. There was no difference between X4 and R5 for time to LF progression (p = 0.925). Estimated risk of LF at 24 months (95% CI) after baseline in X4 and R5 was 10.6% (8.3–12.9) and 9.9% (5.9–14.0), respectively. Age, HCV co-infection, diabetes, HIV-duration, HIV-RNA>100.000 cp/mL, antiretroviral therapy exposure were associated with LF progression at multivariate analysis.

**Conclusions:**

A slight LF progression over time was observed in HIV-infected patients. No difference was demonstrated for X4 and R5 HIV-1 strains in accelerating LF evolution.

## Introduction

A significant risk of liver fibrosis (LF) has been described in HIV infected patients, even in the absence of other common causes of liver disease, such as HBV or HCV co-infections, drugs use, alcohol abuse, metabolic diseases, and immune-suppression [1–5], thereby suggesting a potential role of HIV itself as a cause of liver damage in vivo. Conversely, the control of HIV RNA levels induced by antiretroviral therapy (ART) appears to slow this process [4–6].

However, the pathogenetic mechanism underlying this association is not yet completely understood, but does probably include either direct or indirect pathways [7]. Among the indirect ones, HIV infection in the gastrointestinal tract amplifies microbial translocation, which can stimulate hepatocytes, Kuppfer cells and Hepatic Stellate Cells (HSC) to produce pro-inflammatory cytokines and chemokines [8–9]. These mediators attract activated lymphocytes and monocytes to the liver further inducing fibrosis [8–9]. Moreover, the state of HIV-associated chronic immune activation associated with CD4 cell loss could also induce intra-hepatic inflammation, thus enhancing liver disease progression [4]. In addition, HIV itself and many antiretroviral drugs may contribute to liver disease by inducing insulin resistance and metabolic syndrome [10].

HIV can also directly promote the fibrosis process by activating the HSCs [11–12]. In fact, HSCs, which play a key role in the pathogenesis of fibrosis, do not express CD4 receptors, but present both HIV CCR5 and CXCR4 co-receptors on their surface. *In vitro* experiments showed that, even in the absence of a productive infection, the HIV-1 gp120 binding to CXCR4 [11] and CCR5 [12] is able to activate HSCs. In addition, the HIV *env* gp120 was demonstrated to induce hepatocyte apoptosis *in vitro* through CXCR4 in the absence of infection [13], thus triggering the pro-fibrotic activity of HSC. Whether X4 or R5 viral strains exert a different pro-fibrogenic effect on HSCs *in vivo* is still unknown [14–15].

Herein, we aimed to evaluate in a large HIV-infected cohort if patients carrying X4 or R5 strains have a different progression of LF over time, after adjustment for other causes of liver disease.

## Patients and methods

### Study population

The ICONA Foundation Study is an observational cohort of HIV-infected individuals who are antiretroviral naïve at the time of enrolment. This cohort was set up in January 1997 and currently consists of more than 14,000 patients from 50 Italian infectious disease units. The ICONA Foundation study has been approved by IRB of all the participating centers (S2 File); sensitive data from patients are seen only in aggregate form. All patients sign a consent form to participate in ICONA, in accordance with the ethical standards of the committee on human experimentation and the Helsinki Declaration (1983 revision). Demographic and socio-behavioral data, initiation and discontinuation dates of each antiretroviral drug, HIV-viral load and CD4 cell count every 3–6 months, AIDS defining diseases according to Centers for Disease Control and Prevention (CDC) criteria as well as non-HIV related diseases and death are recorded for all enrolled patients. Haematochemical data, including liver function parameters, are also available at 3–6 months intervals. Further details are available at http://www.fondazioneicona.org/. In the present study, all HIV-infected patients from the ICONA cohort with an available determination of HIV-1 co-receptor tropism (CRT) and at least one-year follow-up were included in the study.

### Co-receptor tropism

Co-receptor tropism (CRT) was assessed and reported by each participating center. Accepted methods for tropism assignment were the gp120 sequencing on plasma RNA and g2p algorithm (http://co-receptor.bioinf.mpi-inf.mpg.de/), setting the false positive rate (FPR) at 10%, according to the 2011 European Guidelines [16], and/or enhanced sensititvity Trofile Coreceptor Tropism Assay (ESTA) (Monogram Biosciences, San Francisco, CA, USA) [17]. Therefore, patients were classified into two groups: R5 (FPR>10% by g2p, or R5-tropic by Trofile) or X4 patients (FPR ≤10% by g2p, or X4-tropic or R5X4-tropic viruses by Trofile assay).

### Statistical analysis

Liver fibrosis was prospectively assessed by means of Fibrosis-4 (FIB-4) Index for Liver Fibrosis at the time of CRT testing and at 3–6 month-intervals up to the last available determination. FIB-4 was calculated using Sterling’s formula, as follows: age [years] × AST [IU/L]/platelet count [expressed as platelets × 10^9^/L] × (ALT1/2[IU/L]). Patients with a FIB-4 score >3.25 or clinical diagnosis of severe liver disease at baseline were excluded from the analysis.

LF progression was defined as an increase of the absolute score or the transition to a higher fibrosis stratum and /or the occurrence of liver-related clinical events, including hospitalization.

Baseline date was defined as the date of tropism test. Patients’ characteristics were compared between the two groups (X4 and R5 patients) using Chi-squared tests for categorical data and Kruskall Wallis tests for continuous data.

A mixed effects linear model was fitted to determine longitudinal trends in FIB-4 score. Time to LF was estimated using Kaplan Meier method stratified by CRT.

Univariable and multivariable Cox regression and ordinal logistic regression were fitted to determine factors associated with LF progression. Potential cofounders measured at baseline included age, education, nationality, mode of HIV transmission, CD4, nadir CD4, HIV-RNA viral load, AIDS diagnosis, ART exposure, diabetes, calendar year of tropism test, HBV/HCV co-infection status. Factors associated with a p-value<0.10 at the univariable analyses were included in the multivariate model.

A subgroup analysis was performed including only patients naïve for ART.

All statistical analyses were performed using SAS (version 9.4; SAS Institute, Cary, North Carolina, USA).

## Results

### Baseline characteristics

A total of 1,137 HIV-infected patients were included with median (IQR) follow-up duration of 34 (14–58) months for X4 and 36 (18–59) for R5, respectively. A total of 846 patients (74.4%) had CRT assessed by gp120 sequencing, while the phenotypic assay was used in the remaining 291 cases (25.6%).

Two hundred forty-nine patients (22%) carried a X4 virus, while the remaining 888 patients had a R5 strain (78%). Baseline characteristics, overall and stratified by tropism are shown in **Table 1.** A total of 668 patients (58.8%) were ART naïve at the time of CRT determination. Patients with X4 virus were more frequently females, intravenous drug users and older compared to R5 patients [40 (IQR: 32–47) and 37 (IQR: 30–45) years, respectively)]. A lower CD4 nadir was observed in X4 patients compared to their R5 counterparts: 284 (IQR: 119–448) cells/mmc and 397 (IQR: 249–538) cells/mmc, respectively.

**Table 1:** Characteristics of patients according to estimated tropism.

	Predicted tropism			
	R5	X4	p-value	Total
	N = 888	N = 249		N = 1137
***Gender*, *n(%)***			0.558	
Female	181 (20.4%)	55 (22.1%)		236 (20.8%)
***Age*, *years***			0.020	
Median (IQR)	37 (30, 45)	40 (32, 47)		38 (31, 46)
***Mode of HIV Transmission*, *n(%)***			0.030	
PWID	51 (5.7%)	27 (10.8%)		78 (6.9%)
MSM	416 (46.8%)	109 (43.8%)		525 (46.2%)
Heterosexual contacts	359 (40.4%)	92 (36.9%)		451 (39.7%)
Other/Unknown	62 (7.0%)	21 (8.4%)		83 (7.3%)
***Nationality*, *n(%)***			0.292	
Italian	727 (81.9%)	211 (84.7%)		938 (82.5%)
***HBsAg*, *n(%)***			0.114	
Negative	802 (90.3%)	235 (94.4%)		1037 (91.2%)
Positive	12 (1.4%)	1 (0.4%)		13 (1.1%)
Not tested	74 (8.3%)	13 (5.2%)		87 (7.7%)
***HCVAb*, *n(%)***			0.723	
Negative	778 (87.6%)	216 (86.7%)		994 (87.4%)
Positive	59 (6.6%)	20 (8.0%)		79 (6.9%)
Not tested	51 (5.7%)	13 (5.2%)		64 (5.6%)
***Hepatitis co-infection*, *n(%)***			0.376	
No	735 (82.8%)	212 (85.1%)		947 (83.3%)
Yes	71 (8.0%)	21 (8.4%)		92 (8.1%)
Not tested	82 (9.2%)	16 (6.4%)		98 (8.6%)
***AIDS diagnosis*, *n(%)***			0.152	
Yes	80 (9.0%)	30 (12.0%)		110 (9.7%)
***Time from HIV diagnosis to date of CRT test*, *years***			0.933	
Median (IQR)	1.71 (1.66, 1.78)	1.72 (1.66, 1.77)		1.72 (1.66, 1.78)
***CD4 count*, *cells/mmc***			0.066	
Median (IQR)	439 (291, 602)	404 (201, 580)		433 (275, 587)
***CD4 count nadir*, *cells/mmc***			< .001	
Median (IQR)	397 (249, 538)	284 (119, 448)		369 (205, 521)
***CD8 count*, *cells/mmc***			0.780	
Median (IQR)	907 (640, 1254)	870 (579, 1305)		905 (624, 1275)
***Viral load*, *log10 copies/mL***			0.887	
Median (IQR)	4 (3, 5)	4 (2, 5)		4 (3, 5)
***ART exposure status***			0.851	
Naive	523 (58.9%)	145 (58.2%)		668 (58.8%)
Experienced	365 (41.1%)	104 (41.8%)		469 (41.2%)
***Diabetes*, *n(%)***			0.711	
Yes	18 (2.0%)	6 (2.4%)		24 (2.1%)
***Method used for tropism testing***			0.152	
Trofile	236 (26.6%)	55 (22.1%)		291 (25.6%)
Geno2pheno	652 (73.4%)	194 (77.9%)		846 (74.4%)
***Follow-up (months)***			0.694	
Median (IQR)	34.1 (14.0, 57.6)	36.2 (17.7, 58.8)		34.5 (14.8, 58.1)

Chi-square or Kruskal-Wallis test were applied as appropriate

Legend: IQR: interquartile range; PWID: People Who Inject Drugs; MSM: men who have sex with men; CRT: co-recptor tropism; ART: antiretroviral therapy

### Liver fibrosis progression according to CRT

Similar baseline FIB-4 scores were measured for patients harbouring X4 and R5 strains [1.01 (0.93–1.09) vs 0.97 (0.93–1.01), respectively]. Mean FIB-4 slope over time was similar in X4 and R5 patients: -0.04/5 years (-0.40, 0.33) versus 0.06 (-0.14, 0.26), p = 0.644, respectively.

A total of 218 (20%) LF progression events were observed during the study period.

**Fig 1:**
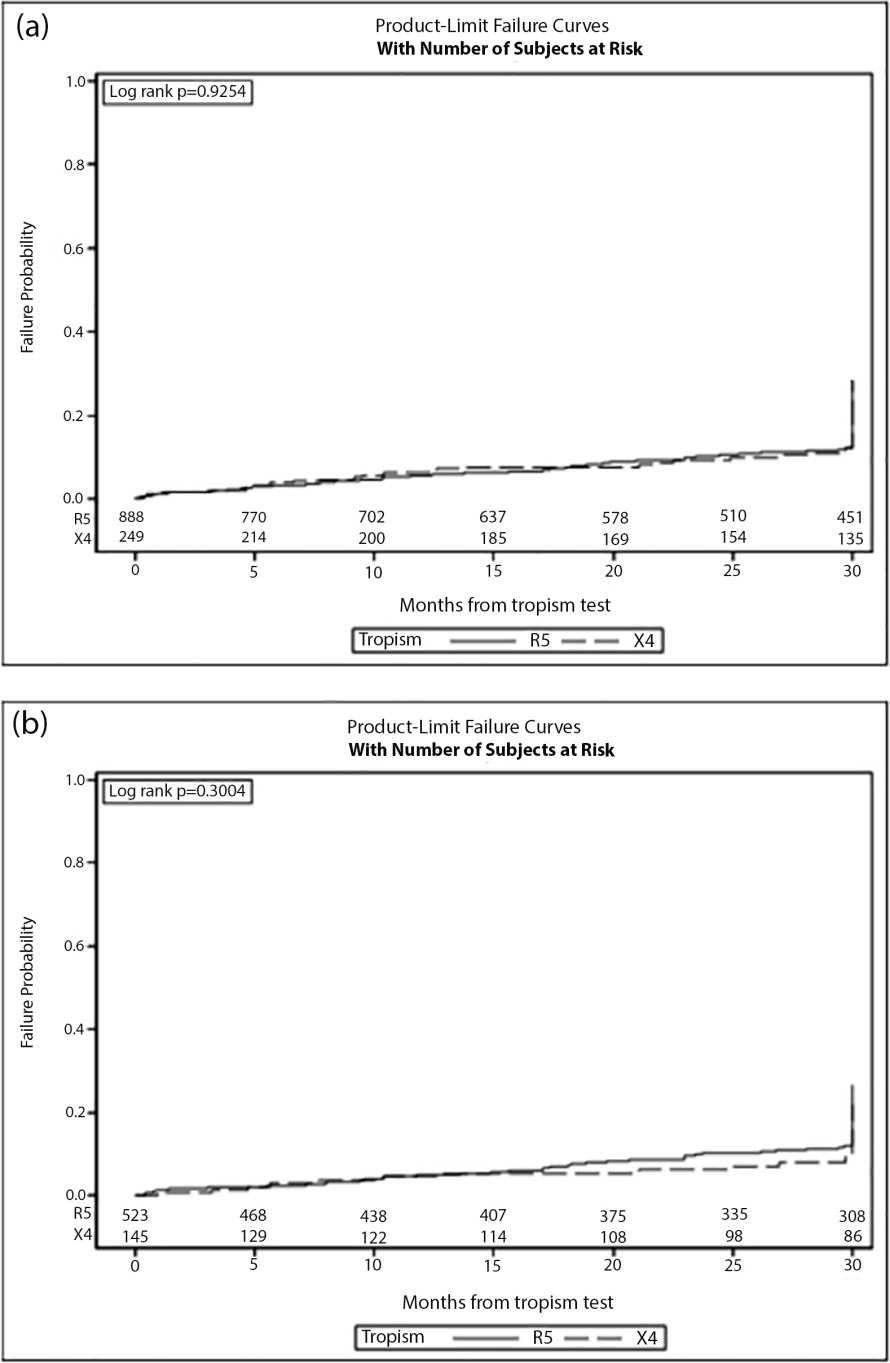
Kaplan-Meier estimates of liver fibrosis progression according to HIV tropism **(a)** in the whole cohort (N = 1,137), and **(b)** in the subgroup of ART-naive patients (N = 668).

There was no difference between X4 and R5 for time to LF progression (p = 0.925). Estimated risk of LF at 24 months (95% CI) after baseline in X4 and R5 was 10.6% (8.3–12.9) and 9.9% (5.9–14.0), respectively (**Fig 1A**).

In the multivariate analyses (Cox regression), after adjusting for potential confounders, no differences in LF progression were observed between the two groups [X4 vs R5 adjusted aRH = 0.89 (95% CI: 0.62, 1.28), p = 0.533]. Similar findings were observed in the ordinal logistic regression after adjustment for potential confounders [aOR X4 vs R5 1.10 (95%CI: 0.75–1.63,p = 0.624] (**Table 2**).

Factors associated with LF at multivariate analysis included: age, HCV infection, nadir CD4, HIV-RNA viral load>100,000 copie/mL, diabetes, exposure to ART (**Table 3**).

**Table 2:** Ordinal logistic regression model.

Odds ratios from fitting an ordinal logistic regression analysis								
	Fibrosis progression							
	Score = 0	Score = 1	Score = 2	Score> = 3	OR (95% CI)	p-value	OR (95% CI)	p-value
					Unadjusted		Adjusted*	
Tropism	N = 919	N = 100	N = 43	N = 75				
X4—no.(%) (N = 249)	199 (79.9%)	24 (9.7%)	5 (2.0%)	21 (8.4%)				
R5—no.(%) (N = 888)	720 (81.1%)	76 (8.5%)	38 (4.3%)	54 (6.1%)				
X4 vs. R5					1.09 (0.76, 1.54)	0.647	1.10 (0.75, 1.63)	0.624

Ordinal endpoint from 3.25 = +2 points; from 1.45–3.25 >3.25 = +1 point; new diagnosis or hospitalization for SLD = +3 points.

*Adjusted for a number of factors measured at the time of tropism test—mode of HIV transmission, nationality, AIDS diagnosis, B/C co-infection status, calendar year, age, CD4 count, CD4 nadir, HIV-RNA, diabetes, ART exposure status and stratified by clinical site

**Table 3:** Relative hazard of time to FIB4 progression.

	Relative hazards of time to Fib4 progression			
	Unadjusted RH(95% CI)	p-value	Adjusted RH(95% CI)	p-value
***Tropism***				
R5	1.00		1.00	
X4	0.99 (0.70, 1.40)	0.952	0.86 (0.59, 1.26)	0.444
***Mode of HIV Transmission***				
PWID	1.00		1.00	
MSM	0.61 (0.35, 1.05)	0.076	1.05 (0.62, 1.76)	0.776
Heterosexual contacts	0.69 (0.40, 1.19)	0.188	0.86 (0.52, 1.42)	0.868
***Calendar year of test***				
per more recent	1.13 (1.06, 1.20)	< .001	0.76 (0.44, 1.32)	0.324
***Age*, *years***				
per 10 years older	1.44 (1.25, 1.65)	< .001	1.40 (1.20, 1.64)	< .001
***CD4 count*, *cells/mmc***				
<200	1.00		1.00	
201–350	0.53 (0.27, 1.06)	0.074	0.68 (0.29, 1.61)	0.383
>350	0.52 (0.31, 0.89)	0.018	1.07 (0.51, 2.24)	0.850
Unknown	0.65 (0.39, 1.10)	0.106	0.85 (0.23, 3.07)	0.799
***CD4 count nadir*, *cells/mmc***				
<200	1.00		1.00	
201–350	0.67 (0.44, 1.03)	0.071	1.04 (0.61, 1.76)	0.887
>350	0.64 (0.45, 0.92)	0.015	1.00 (0.60, 1.67)	0.986
Unknown	1.15 (0.49, 2.70)	0.756	1.08 (0.39, 2.77)	0.867
***Viral load*, *log10 copies/mL***				
<5000	1.00		1.00	
5000–10000	0.85 (0.33, 2.19)	0.741	1.21 (0.44, 3.34)	0.708
10000–100000	0.99 (0.58, 1.69)	0.972	1.42 (0.79, 2.61)	0.257
>100000	1.46 (0.86, 2.48)	0.158	2.05 (1.13, 3.81)	0.022
Unknown	1.22 (0.79, 1.90)	0.374	1.89 (0.53, 6.80)	0.332
***Aids diagnosis***				
Yes vs. No	1.70 (1.08, 2.67)	0.021	1.65 (0.97, 2.80)	0.066
***ART exposure status***				
Not Naïve	1.00		1.00	
Naïve	0.69 (0.50, 0.94)	0.018	0.63 (0.43, 0.94)	0.024
***Time from HIV diagnosis to date of tropism test***				
per more recent	1.01 (1.01, 1.02)	< .001	1.04 (0.99, 1.09)	0.114
***HCV infection***				
No	1.00		1.00	
Yes	2.23 (1.43, 3.49)	< .001	3.30 (1.92, 5.67)	< .001
Not tested	0.87 (0.33, 2.29)	0.779	0.65 (0.24, 1.73)	0.387
***Diabetes***				
Yes vs. No	3.47 (1.54, 7.78)	0.003	3.63 (1.54, 8.62)	0.003

Adjusted for a number of factors measured at the time of tropism test—mode of HIV transmission, nationality, AIDS diagnosis, B/C co-infection status, calendar year, age, CD4 count, CD4 nadir, HIV-RNA, diabetes, use of lipids/blood pressure lowering drugs, ART exposure status and stratified by clinical site.

### Subgroup analysis in patients naïve to ART

A sub-analysis was carried out including only naïve patients with CRT assessed before the initiation of their first ART regimen (N = 688, of whom 523 R5 and 145 X4 subjects). Again the two groups were similar for baseline characteristics, except for a higher prevalence of IDUs and a lower nadir CD4 in X4 compared to their R5 counterpart. By the Kaplan Meier analysis, a reduced risk for fibrosis progression was observed in X4 compared to R5 patients but this difference was not statistically significant (p = 0.30) (**Fig 1B**). Similarly, by fitting an ordinal logistic regression model, the adjusted OR in X4 versus R5 was 0.69 (95% CI: 0.39, 1.22; p = 0.199). Moreover, in the Cox model, the unadjusted relative hazard for fibrosis progression for X4 patients was 0.69 (95% CI: 0.41, 1.17). However, after adjustment for other baseline variables (including mode of HIV transmission, nationality, AIDS diagnosis, B/C co-infection status, calendar year, age, CD4 count, CD4 nadir, HIV-RNA, and diabetes) the aRH resulted 0.52 (95% CI: 0.29, 0.93), reaching statistical significance (p = 0.029).

## Discussion

CCR5 and CXCR4 co-receptors, belonging to the family of beta-chemokines receptors, in association with the main CD4 receptor, play a fundamental role for entry of HIV into target cells of the immune system including T lymphocytes, monocyte-macrophages and dendritic cells [18]. Their presence has also been demonstrated in many different body sites including the liver [7, 11–12, 18]. *In vitro* experiments performed by diverse research groups showed that, even in the absence of a productive infection, the HIV-1 gp120 binding to CXCR4 [11] and CCR5 [12] is able to activate the Hepatic Stellate Cells, thus promoting the fibrogenetic process. If this mechanism has a clinical relevance is still unknown.

In a previous study [15], in a group of 105 HIV/HCV co-infected patients, treated with ART and tested for CRT on proviral DNA, we retrospectively evaluated the evolution of fibrosis as determined by means of APRI and FIB-4 surrogate scores starting from the first anti-HCV positive testing to the date of CRT assessment. No difference was found in the liver fibrosis evolution between the two groups of R5 and X4 patients. Similarly, in a French study [14] investigating the influence of HIV tropism (phenotypic assay) on liver stiffness (transient elastography) in 172 HIV/HCV co-infected patients, the prevalence of X4 viruses did not significantly differ in patients with mild (F0–F2) vs severe fibrosis (F3–F4). As both these studies contradicted *in vitro* results and were based on a limited sample size, we feel that a larger and prospective clinical study was needed to validate their findings. Herein, we aimed to prospectively investigate whether HIV-1 CRT impacts the progression of liver fibrosis over time in a very large group of HIV positive patients tested for CRT, most of whom were not HBV or HCV co-infected, with a median follow-up time of three years.

An overall 10% risk of LF progression by 24 months was observed in our cohort, thus confirming previous observations [1–5] that claimed for a significant risk of liver fibrosis in HIV infected patients, even in the absence of other common causes of liver disease.

No difference was demonstrated for X4 and R5 HIV-1 strains in accelerating LF evolution, with consistent findings in all our analyses after adjustment for potential confounders. However, when considering exclusively ART naive patients, a reduced risk of liver fibrosis in X4 compared to R5 patients was observed, even if this difference reached statistical significance only by the multivariable Cox regression analysis. This finding deserves further investigations. However, our result might rely on some *in vitro* experiments. In fact, Bruno et al. [12] found that while exposure of HSCs to increasing concentrations of a recombinant R5-derived (CN54) gp120 increased cell migration in a dose-dependent fashion, the IIIB gp120 (from a X4-strain) induced HSC migration less effectively and only at very high concentrations. In the same paper, R5-derived gp120 also led to a significant increase in the secretion and gene expression of MCP-1 (pro-inflammatory chemokine monocyte chemoattractant protein-1), and in an increased gene expression of TMP-1 (tissue inhibitor of metalloprotease-1) and IL-6 (interleukin-6). Moreover, both X4 and R5 gp120 demonstrated to promote the production TGF-ß1, which is a key cytokine released by HSCs as a response to a liver injury [19]. However, higher levels of TGF-ß1 were found by Gupta et al [20] after exposure of human immortalised HSC line (LX2) cells with culture supernatants of human PBMCs infected with a R5-gp120 rather than with a X4 one.

It is known that X4 and R5 strains entail different remarkable virological and immunological effects [18], as it was also confirmed in our cohort where a lower nadir CD4 count applied to X4 patients. Therefore, if we hold true that HIV-1 *env* has a primary direct role in causing fibrosis *in vivo*, it seems difficult to accept that differences between X4 and R5 viruses do not reflect a clear divergent evolution of liver fibrosis in subjects harboring one or other of the strains. This apparent contradiction might be explained by the fact that although R5 and X4 modulate different aspects of HSC biology, common final pathways are on the basis of liver fibrosis pathogenesis, independently on the first causative trigger [20]. As we were able to demonstrate a greater effect on liver inflammation and fibrosis of R5 variants only in naïve patients, we can hypothesize that this difference is not striking and is probably attenuated by the contemporary presence of many other potential confounders, especially in ART-experienced patients.

In fact, progression of liver fibrosis in our population was clearly driven by other classical co-factors including age, HCV co-infection, and diabetes. Our results also confirm that HIV itself is involved in the development of liver fibrosis: in fact, not only the duration of HIV infection, but also HIV viremia were associated with LF: in particular, high (>100.000 copies/ml) levels of HIV viremia determined a two-fold increase of the risk of LF, which is in agreement with previous papers [1–5]. Indirectly, the immune activation state induced by HIV replication (which is due to multiple mechanisms including response to CD4 cell depletion and microbial translocation), could be responsible for an increased pro-inflammatory stimulus on liver parenchyma which, in turn, leads to an accelerated fibrotic process [4;21].

Unlike previous studies [1–5], in our experience, ART exposure was associated with risk of LF progression; however, as CRT determination was performed on plasma samples, only viremic patients, including those in virological failure, were considered. Therefore, we should conclude that the beneficial effects of ART on viral suppression remedy its potential liver toxicity.

We acknowledge that our study presents some limitations. Firstly, the heterogeneity of CRT determination could have influenced our findings, even if our results did not change when adjusted for the method used to assess HIV tropism. In the majority (74%) of our patients, CRT was assessed by a genotypic method. Based on the HIV-1 gp120 sequence, the geno2pheno interpretation furnishes a FPR (false positive rate) which is a percentage score indicating the likelihood that a V3 sequence is falsely predicted as CXCR4-using patients. Therefore, patients were classified as X4 and R5 based on an arbitrary cut-off of 10% FPR as suggested by current guidelines for tropism testing [16]. As the presence of a dual/mixed population of R5 and X4 viruses is not defined with this method, a misclassification of strains cannot be excluded.

In our study, CRT was determined on plasma samples; while a direct fibrogenic action of HIV envelope protein has been demonstrated in HSCs [12]; nonetheless, whether X4 or R5 strains have a different weight on the overall fibrogenetic process is reasonably also dependent on a different expression of CCR5 and CXCR4 receptors in liver tissue, which can change over the course of the infection and according to different clinical conditions. For this purpose, only studies based on liver biopsies could help to clarify this issue. Moreover, if ART initiation can induce a co-receptor shift is controversial [22]; this might partially explain differences we found in ART treated and naïve patients.

Liver fibrosis was assessed by means of a surrogate marker (FIB-4 score) which has a limited sensitivity and specificity compared to other methods, such as transient elastography; however, the use of FIB-4 for the evaluation of fibrosis has been validated and widely used in previous studies, also in the population of HIV infected patients [23]. Finally, other unmeasured confounding can exist which has not been accounted for.

In conclusion, we confirm the potential risk for liver fibrosis associated with HIV infection; moreover, our study demonstrates a similar propensity of X4 and R5 strains to induce liver inflammation and, consequently, to accelerate the fibrosis process in HIV-infected patients.

## Supporting information

S1 File Dataset. (XLSX)

S2 File List of ethic committees. (DOCX)
